# Ovarian Fibromatosis in Puberty: A Long-Term Battle

**DOI:** 10.7759/cureus.89454

**Published:** 2025-08-05

**Authors:** Melanie C Norton, Doaa Ahmed, Mahmoud Awaly, Brianna Cloke

**Affiliations:** 1 Gynaecology, Guy's and St Thomas' NHS Foundation Trust, London, GBR

**Keywords:** hormones in gynecology, ovary, pediatrics, puberty, puberty onset, torsion

## Abstract

Ovarian fibromatosis (OF) is a rare, benign condition that often mimics malignancy, leading to unnecessary oophorectomies. We report the case of a 12-year-old girl presenting with acute right lower abdominal pain and vomiting, with a history of intermittent abdominal pain since age seven. Imaging revealed an enlarged, avascular right adnexa with a solid lesion. Despite normal adrenal imaging, her hyperandrogenism raised concerns for an underlying endocrine pathology. Multidisciplinary team discussions led to surgical exploration, revealing ovarian torsion with an abnormal ovary, necessitating oophorectomy. Histopathology confirmed OF. Postoperatively, her androgen levels normalized; however, prolonged androgen exposure resulted in persistent hyperandrogenic features. This case highlights the diagnostic challenge of OF, emphasizing its link to massive ovarian oedema and disrupted steroidogenesis. Recognizing OF preoperatively is critical to prevent overtreatment. A conservative approach with ovarian biopsy should be considered to avoid unnecessary oophorectomy in young patients.

## Introduction

Ovarian fibromatosis (OF) is a rare, benign, non-neoplastic condition characterized by diffuse ovarian enlargement due to stromal proliferation of collagen-producing spindle cells. It predominantly affects young females and often presents with abdominal pain, menstrual irregularities, or a solid adnexal mass. In some cases, hyperandrogenic features such as hirsutism and virilization may occur due to disrupted ovarian steroidogenesis [1,2]. Although histologically benign, OF often mimics ovarian malignancy on imaging and intraoperative inspection, which can lead to unnecessary oophorectomy [3]. The average age of onset is around 25 years, likely reflecting the dynamic hormonal and structural changes occurring in the ovary during adolescence [4]. 

The exact pathogenesis of OF remains poorly understood. It is postulated to be a reparative stromal response to chronic or intermittent ovarian torsion, prior inflammation, or hormonal stimulation. The fibrotic process may entrap normal ovarian follicles and, in some cases, is associated with massive ovarian edema (MOE), further complicating diagnosis [5]. Here, we present a rare case of OF in a 12-year-old girl who developed longstanding endocrinologic abnormalities and altered pubertal progression, highlighting the diagnostic and therapeutic challenges associated with this condition.

## Case presentation

**Figure 1:**
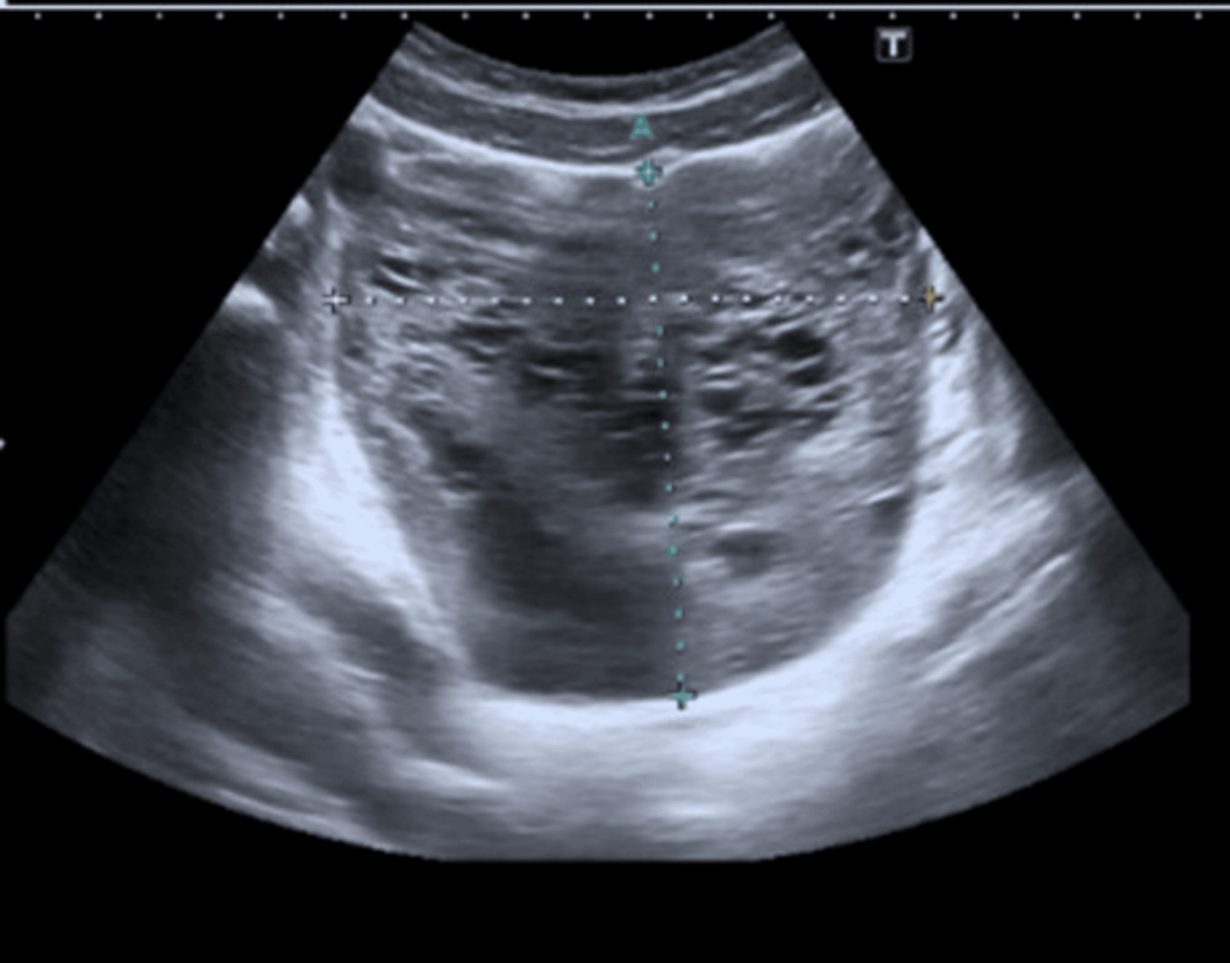
Transabdominal ultrasound scan showing adnexal mass

**Figure 2:**
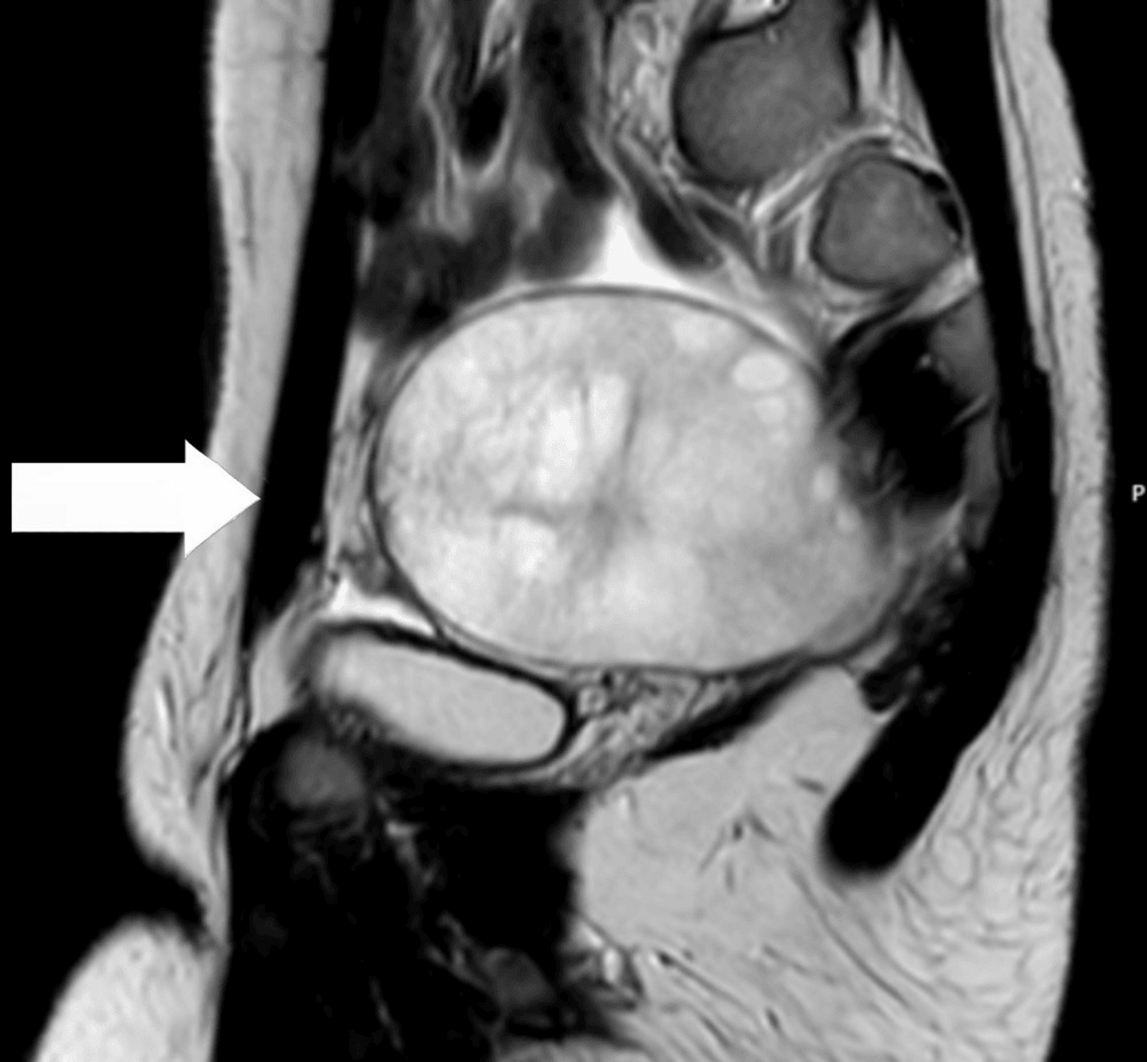
Pelvic MRI showing adnexal mass in sagittal section

**Figure 3:**
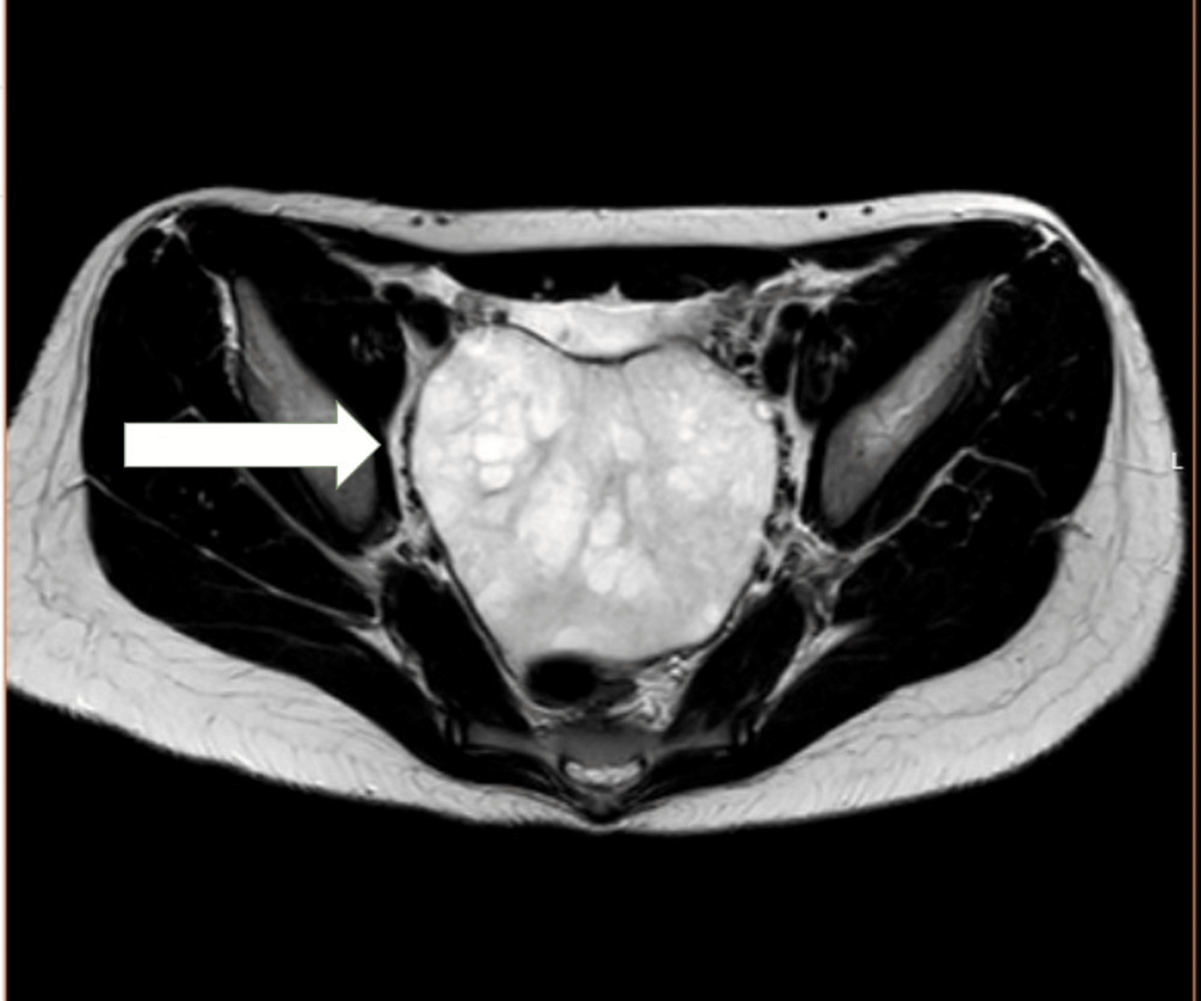
Pelvic MRI showing adnexal mass in axial (transverse) section

A 12-year-old girl presented to the emergency department with acute right lower abdominal pain, accompanied by nausea and vomiting. She reported having had similar intermittent pain since the age of 7. Her clinical picture raised suspicion for ovarian torsion, and she was promptly evaluated by the on-call gynaecology team. Her history revealed a longstanding pattern of intermittent abdominal pain since the age of seven, with multiple prior emergency department visits yielding no abnormalities. She had no known medical comorbidities. On examination, the patient was haemodynamically stable with localized right lower quadrant tenderness, but without peritoneal signs. Signs of hyperandrogenism, including hirsutism and acne, were also noted. Transabdominal ultrasound showed an enlarged right adnexa with a heterogeneous cystic structure and absent Doppler flow to the ovary, raising suspicion for torsion (Figure 1). Pelvic MRI further demonstrated a well-defined solid lesion with benign features; however, malignancy (e.g. sarcoma) could not be definitively excluded (Figures 2, 3). Bilateral adrenal glands were normal. Laboratory workup revealed elevated testosterone, androstenedione, oestradiol, and cortisol levels, while tumour markers (cancer antigen 125 (CA-125), lactate dehydrogenase (LDH), alpha-fetoprotein (AFP)) were within normal limits.

**Figure 4:**
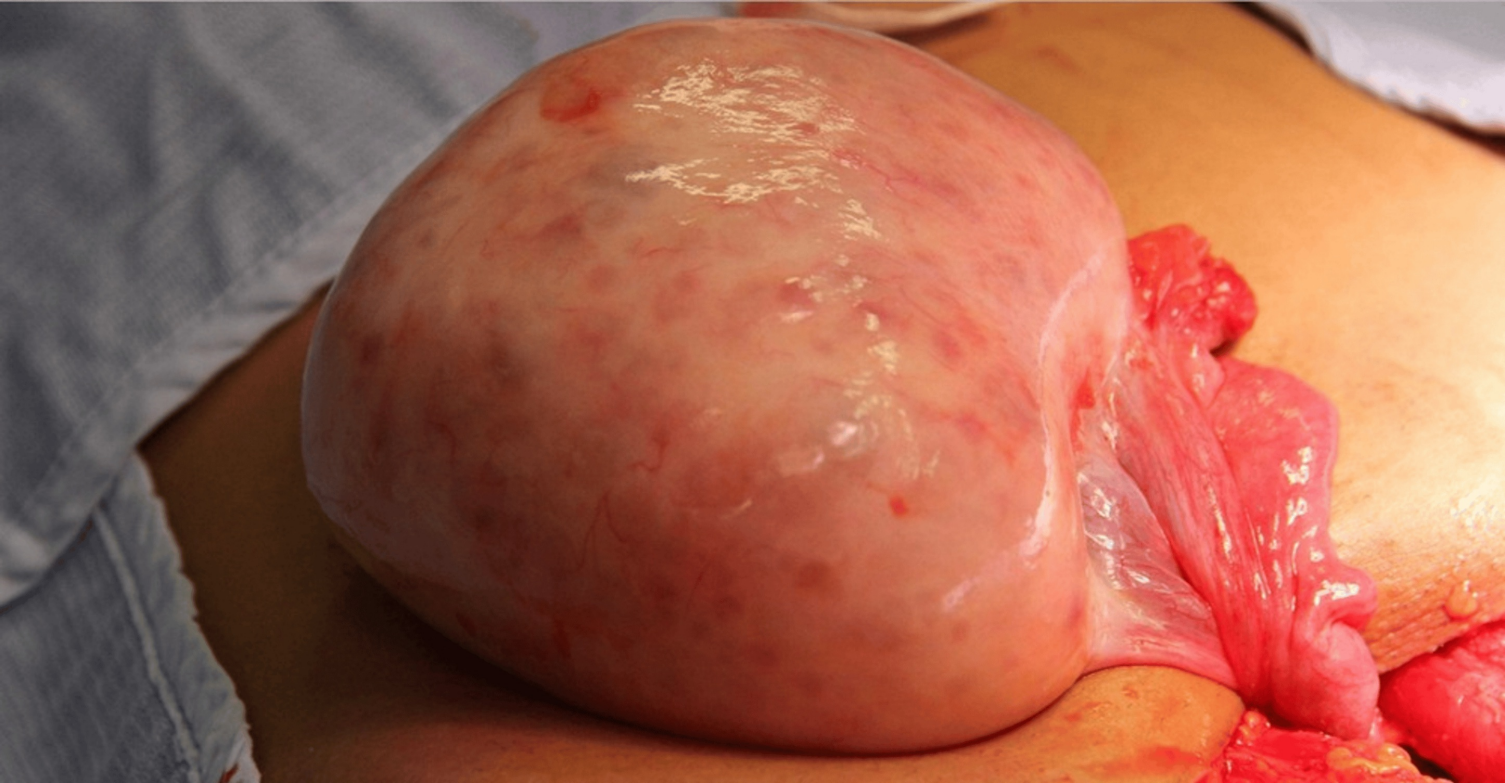
Intraoperative findings showing enlarged and abnormal ovary

The case was discussed in a multidisciplinary team (MDT) meeting involving paediatric endocrinology, paediatric and benign gynaecology, and gynae-oncology specialists. Due to ongoing concern for ovarian torsion with an unclear underlying lesion, the patient underwent a Pfannenstiel laparotomy. Intraoperatively, the right ovary was found to be torted and grossly abnormal, necessitating a right oophorectomy (Figure 4). The left ovary appeared normal and was not biopsied. No ascites or other pelvic abnormalities were observed.

Histopathological analysis confirmed OF. The ovarian stroma exhibited dense fibrosis with collagen-producing spindle cells and interspersed luteinized stromal cells. Normal ovarian follicles were seen entrapped within the fibrous stroma, and no features of malignancy were identified. It was hypothesized that the patient had developed massive ovarian oedema due to chronic intermittent torsion, which later progressed to OF through a reparative inflammatory process.

The patient’s postoperative recovery was uncomplicated. Over the following months, her androgen levels gradually normalized. However, the prolonged exposure to elevated androgens had already resulted in persistent hyperandrogenic features, including hirsutism, deepened voice, and a prominent larynx. She remains under regular follow-up with the Paediatric Gynaecology Team for ongoing endocrine and developmental monitoring. Additionally, she is receiving ENT follow-up for voice changes and a prominent thyroid cartilage (laryngeal prominence) associated with prolonged androgen exposure.

## Discussion

This case illustrates the diagnostic complexity of ovarian fibromatosis, particularly in young patients. OF remains a rare and often under-recognized entity, frequently misdiagnosed as an ovarian neoplasm due to its imaging characteristics and solid appearance during surgery. In children and adolescents, the diagnostic challenge is even greater, given the lower index of suspicion for fibrotic ovarian conditions [1].

The relationship between massive ovarian oedema (MOE) and OF is well-documented in the literature, with some authors proposing that they exist on a pathological continuum [6]. In this case, intermittent torsion likely led to MOE, which evolved into OF through a chronic stromal reparative response. The associated luteinization of stromal cells may explain the patient’s hyperandrogenic symptoms.

Given the benign nature of OF, conservative surgical approaches are preferred. Where feasible, ovarian-sparing procedures and intraoperative frozen section biopsy should be considered to confirm the diagnosis and avoid unnecessary oophorectomy. It is important to consider this as a differential diagnosis when interpreting imaging (such as MRI) [3,7]. Unfortunately, in our case, the grossly abnormal appearance of the ovary combined with diagnostic uncertainty led to a more radical approach.

Early recognition of OF is essential, especially in adolescents, to preserve reproductive and endocrine function. In cases such as these, signs of hyperandrogenism in adolescent females should prompt further investigations, as these can be linked to OF [5]. Multidisciplinary involvement and heightened awareness can aid in tailoring management strategies that prioritize organ preservation.

## Conclusions

OF should be considered in the differential diagnosis of solid ovarian masses, particularly in young patients presenting with features of torsion and hyperandrogenism. Despite its benign pathology, its tumour-like appearance often leads to overtreatment. Conservative management, including intraoperative biopsy, should be pursued when possible to preserve ovarian function and minimize long-term endocrinologic consequences. This case highlights the importance of preoperative suspicion and intraoperative caution to prevent unnecessary oophorectomy in paediatric patients.
